# Laparoscopic Inguinal Hernia Repair in the Obese Patient Population: A Single-Center Five-Year Experience

**DOI:** 10.7759/cureus.48265

**Published:** 2023-11-04

**Authors:** Nikolaos Pararas, Anastasia Pikouli, Messaoud Bounnah, Yousef Zenah, Abdulkarim M Alkadrou, Dimitrios Papakonstantinou, Dionysios Dellaportas, Emmanouil Pikoulis

**Affiliations:** 1 General Surgery, Dr Sulaiman Al Habib Hospital, Alfaisal University, Riyadh, SAU; 2 3rd Surgical Department, Attikon University Hospital, National and Kapodistrian University of Athens School of Medicine, Athens, GRC; 3 General Surgery, Dr. Sulaiman Al Habib Hospital, Riyadh, SAU; 4 3rd Surgical Department, Attikon University Hospital, National and Kapodistrian University of Athens, School of Medicine, Athens, GRC

**Keywords:** inguinal hernia, totally extraperitoneal repair (tep), transabdominal preperitoneal repair (tapp), laparoscopic inguinal hernia repair, obesity

## Abstract

Purpose

The objective of the present study is to evaluate the distribution of the transabdominal preperitoneal (TAPP) and the totally extraperitoneal repair (TEP) procedures among the obese and non-obese patient populations, to show how obesity impacts daily practice by reviewing the experience of a single center, and finally, to assess the outcomes of the operations.

Methods

All patients who underwent elective, minimally invasive inguinal hernia repair in our hospital from January 2017 to January 2022 were included in the present study. The data that were analyzed were patient demographics, each individual patient's American Society of Anesthesiology (ASA) score, the minimally invasive technique (TAPP or TEP) utilized, the body mass index (BMI), and other comorbidities such as underlying diabetes, hypertension, and smoking status.

Results

A total of 109 patients were included in the present analysis, of which 81 (74.3%) underwent elective TEP repairs while 28 (25.7%) underwent elective TAPP repairs. Overall, 39 (35.7%) patients were included in the obesity subgroup with an average BMI of 35.4 ± 4.9, with a range from 30.1 to 52.7, and 70 (64.3%) were included in the non-obese subgroup, with an average BMI of 23.2 ± 3.3 and a range from 16.2 to 29.7.

Conclusions

The laparoscopic approach to the inguinal hernia repair in obese patients has similar outcomes as an open approach regarding the 30-day events, in the hands of experienced surgeons with the advantages of the laparoscopic approach vs. the open one.

## Introduction

Over 20 million patients worldwide are estimated to undergo elective inguinal hernia repairs every year [1,2]. The tension-free Lichtenstein repair is the most commonly performed procedure with low complication and recurrence rates [3]. Since the early 1990s when the technique was first published, it has evolved, with new laparoscopic approaches, such as the transabdominal preperitoneal (TAPP) and the totally extraperitoneal repair (TEP), emerging to further enhance patient outcomes [4-10]. Compared to the conventional Lichtenstein technique, the minimally invasive approaches seem to be associated with a reduced risk of early postoperative pain, wound-related infections, chronic pain, and earlier return to work and daily activities compared to the open approach [11]. The advantage of the TEP repair is the non-violation of the peritoneal cavity with the procedure performed entirely in the preperitoneal space [12]. On the contrary, the TAPP repair necessitates entry into the peritoneal cavity, providing a wide operative field and the opportunity to detect unsuspected contralateral hernias [13,14]. Previous studies and meta-analyses yielded conflicting results in the head-to-head comparison of TEP with TAPP, with proponents of the former arguing that it is associated with less postoperative pain while TAPP involves a less steep learning curve [15-21].

The increasing population of obese patients around the world requires a reevaluation of the two minimally invasive techniques in this particular subset of patients. The relationship between obesity and inguinal hernia occurrence and recurrence still remains controversial. Several studies have demonstrated a protective effect of being overweight and obese on the incidence of primary groin hernia [22-25]. Others have reported a linear association between obesity and higher risks of postoperative complications and hernia recurrence following ventral and inguinal hernia repairs [26-29].

The purpose of the present study is to explore how obesity impacts daily practice by reviewing the experience of a single center, to evaluate the distribution of the TAPP and TEP methods among the obese and non-obese patient populations, and to assess relevant postoperative outcomes.

This article was previously posted to the Research Square preprint server on July 15, 2022.

## Materials and methods

Consecutive patients undergoing elective minimally invasive inguinal hernia in our hospital were included from January 2017 to January 2022. Inclusion criteria were patients operated electively from January 2017 to January 2022, patients over 18 years old, patients with unilateral or bilateral uncomplicated inguinal hernias, and patients with recurrent inguinal hernias previously operated with an open technique. Exclusion criteria were patients who underwent emergency surgery, patients with complicated inguinal hernias (not reducible or strangulated), patients with recurrent inguinal hernias previously operated with the laparoscopic approach, patients who, due to medical reasons, could not undergo general anesthesia and patients with no follow-up. For each patient case, data of interest were the minimally invasive technique utilized, patient demographics, individual patient American Society of Anesthesiology (ASA) score, the body mass index (BMI), underlying hypertension and diabetes, and smoking status. Patients were separated into an obese and a non-obese patient subgroup using a BMI cut-off value of 30. Outcomes of interest were the length of the surgical procedure, the postoperative length of hospital stays, and postoperative morbidity and recurrence rates.

In cases of TEP inguinal hernia repair, a conventional 10 mm laparoscopic port was placed above the posterior rectus sheath laterally to the umbilicus preperitoneal space, which was expanded carefully using the endoscope. Standard 5 mm ports were entered in the midline, as per convention, and the preperitoneal dissection was carried to the space of Retzius under direct vision. After the reduction of the hernia sac was complete, a 10x15 cm microporous three-dimensional polypropylene mesh was inserted and was fixated with only a single tacker in the pubic bone. The air in the preperitoneal space was then slowly evacuated under direct vision to ensure that no slippage of the mesh ensued.

In the cases of TAPP inguinal hernia repair, entry into the peritoneal cavity was achieved via the open Hasson technique to insert the 10 mm camera port. After that, two 5 mm ports were inserted in the right and left mid-clavicular lines, just inferior to the level of the umbilicus. The preperitoneal space was entered at the anterior superior iliac spine level, two finger breadths medially, and was carefully dissected until the parietalization of the hernia was complete. Mesh insertion and fixation were identical to TEP cases, and closure of the peritoneal defect at the end of the operation was achieved using absorbable tackers.

Two senior surgeons experienced in both techniques performed all procedures, having performed over 300 lifetime minimally invasive inguinal hernia repairs each. The choice of the procedure (TAPP or TEP) applied to each patient was according to each surgeon's preference. Following hospital discharge, patients were reassessed after a 30-day interval, at which point patient follow-up ended. Ethical committee approval was obtained for this study from the hospital’s Ethical Committee and informed consent was taken from all patients to be included in the current study.

Statistical analysis

All analyses performed used the Statistical Package for Social Sciences (SPSS) version 20.0 (IBM Corporation, Redmond, NY). The chi-square test and Fischer’s exact test were utilized for comparisons between categorical variables. The non-parametric Mann-Whitney-U test was used for comparison between continuous variables. A p-value less than 0.05 was considered statistically significant throughout the analysis.

## Results

A total of 109 patients were included in the present analysis, of which 81 (74.3%) underwent elective TEP repairs while 28 (25.7%) underwent elective TAPP repairs. Overall, 39 (35.7%) patients were included in the obesity subgroup, with an average BMI of 35.4 ± 4.9, with a range from 30.1 to 52.7, and 70 (64.3%) were included in the non-obese subgroup, with an average BMI of 23.2 ± 3.3 and a range from 16.2 to 29.7. When the two subgroups were compared, the obese patient subgroup demonstrated a statistically significant increase in the number of diabetic patients, with a significantly increased predilection toward the utilization of the TEP operative method (89.7% in the obese patient subgroup versus 70% in the non-obese, p=0.01). No statistically significant differences were found regarding ASA score, smoking, and hernia bilaterality. Operative time was equivalent between the compared groups (Table 1), with complication rates being higher in the non-obese subgroup (5.1% versus 12.9% in the non-obese subgroup). However, this finding did not attain statistical significance (p=0.32). The length of hospital stay was marginally increased in the obese subgroup (1.7 ± 0.8 versus 1.5 ± 1.9 in the non-obese subgroup), with the finding being statistically significant (p=0.03).

**Table 1:** Characteristics of the included patient population BMI: body mass index; ASA Score: American Society of Anesthesiologists score; TEP: totally extraperitoneal laparoscopic inguinal hernia repair with mesh; TAPP: transabdominal preperitoneal inguinal hernia repair with mesh

	Total / Mean ± SD (n=109)	Obese patients (n=39)	Non-obese patients (n=70)	p-value
Age	46 ± 16.8	46.4 ± 13.9	45.8 ± 18.3	0.2
Gender (M/F)	105 (96.3%) / 4 (3.7%)	35 (89.7%) / 4 (10.3%)	70 (100%) / 0	0.01
BMI	28.2 ± 6.6	35.4 ± 4.9	23.2 ± 3.3	0.08
ASA score	2 (1-4)	2 (1-3)	2 (1-4)	0.62
Hypertension	26 (23.8%)	8 (20.5%)	18 (25.7%)	0.64
Diabetes	13 (11.9%)	9 (23%)	4 (5.7%)	0.01
Smokers	22 (20.1%)	10 (25.6%)	12 (17.1%)	0.28
TEP / TAPP	81 (74.3%) / 28 (25.7%)	35 (89.7%) / 4 (10.3%)	46 (65.7%) / 24 (34.3%)	0.02
Bilateral hernia	24 (22%)	11 (28.2%)	13 (18.6%)	0.16
Bilateral hernia TEP/TAPP	21 (25.9%) / 3 (10.72%)	10 (28.6%) / 1 (25%)	11 (23.9%) / 2 (8.3%)	0.11
Operative time	98 ± 35.3	96.5 ± 44.1	98.7 ± 29.8	0.59
Length of hospital stay	1 (1-15)	1.7 ± 0.8	1.5 ± 1.9	0.03
Complications	11 (10%)	2 (5.1%)	9 (12.9%)	0.32

No hernia recurrence was encountered in any patient in either group.

## Discussion

The recent European Hernia Society guidelines state that the tension-free Lichtenstein technique and minimally invasive techniques, such as TAPP and TEP, performed by expert surgeons, are the best evidence-based options for inguinal hernia repair [1]. A recent meta-analysis of randomized controlled studies demonstrated that both TEP and TAPP are associated with a reduced risk for postoperative pain and earlier return to work/daily activities compared to open tension-free repair [11]. Hernia recurrence after minimally invasive repair is also comparably low, with cited recurrence rates of up to 2%, for both TEP and TAPP repairs [1-3]. The technique for mesh fixation (self-gripping vs. sutured meshes vs. tacker vs. glue fixation), mesh type, size, overlap extent, sliding hernias, medial or lateral hernia sac, adequate dissection and space creation, operating time, type of anesthesia, participation in a register database, postoperative complications, and center/surgeon volume have previously been identified as risk factors [30].

Postoperative chronic pain after minimally invasive repair has been reported in up to 3% of patients [11]. In our cohort, no cases of chronic pain were reported. Surgeon experience and expertise, variation in technical skills, and hospital volume are key determinants of operative time while TAPP and TEP have been shown to be associated with a sharp learning curve [24]. The European Hernia Society indicated that one hundred TAPP procedures are required to achieve similar results with the Lichnestein repair and that at least 50 cases are required to halve the complication rates [1]. Lau et al. stated that at least 80 TEP repair cases are required to complete the learning curve while Aeberhard et al. reported a significant drop in surgical time duration after one hundred procedures [12,29].

In the present study, both TAPP and TEP procedures were carried out by two qualified minimally invasive surgeons with experience of more than 300 cases for each procedure. Operative time was equivalent between the compared groups; the anesthesia time is included in this time as well (Table 1), with complication rates being higher in the non-obese subgroup (12.9% vs. 5.1% in the obese subgroup), although this finding did not attain statistical significance (p=0.32). There was no conversion to open surgery.

The inguinal hernias repair in obese patients may present many unique challenges to the surgeon due to the increased risks associated with obesity. The excessive preperitoneal fatty tissue and the tendency to develop postoperative complications increase the difficulty of inguinal hernia repair in obese patients. As previously mentioned, obesity appears to have a protective effect on the occurrence of primary groin hernia. Particularly, the obese population has an increased risk of postoperative morbidity [30], which is likely related to the higher incidence of cardiac and metabolic comorbidities that are often present in this patient population [20]. These findings create a diagnostic and therapeutic dilemma in the approach to inguinal hernia repair in the obese population. Both TAPP and TEP techniques have been used in obese patients, but TEP repair is often preferred in this population due to several reasons: a) Avoidance of intra-abdominal access: TEP repair is performed entirely outside the abdominal cavity, eliminating the risk of injury to abdominal organs. This is particularly advantageous in obese patients, as their increased abdominal wall thickness can make TAPP more challenging. b) Reduced postoperative complications: TEP repair has been related to lower rates of postoperative complications, such as wound infections and seromas, compared to TAPP repair in obese patients. c) Better visualization: TEP repair provides a clearer view of the inguinal region due to the lack of intra-abdominal organs, facilitating precise dissection and placement of the mesh. d) Potential for bilateral repair: TEP repair allows for bilateral hernia repair through a unilateral approach, which can be beneficial in cases where both sides are affected.

Nevertheless, in the present study, postoperative complications in the obese group were lower than in the non-obese one, with the majority of the cases having postoperative subcutaneous hematomas, regardless of the laparoscopic approach selected (TEP or TAPP). There was one case of bilateral inguinal hernia operated with TEP that developed necrotizing pancreatitis after the operation, which was attributed to anesthesia-associated medications. There were no surgical site wound infections in any of the patients.

When comparing laparoscopic to open inguinal hernia repairs in obese patients, several authors have reported better outcomes in the laparoscopic group concerning wound morbidity. This difference is likely related to the extensive subcutaneous dissection that often occurs in an open inguinal hernia. In minimally invasive repairs of inguinal hernias, there is often little to no subcutaneous dissection and thus wound-related events might be much less frequent. On the other hand, the large retroperitoneal dissection necessary in a laparoscopic inguinal hernia repair can be exceptionally challenging in an obese patient and might limit the general improvements in outcomes in this patient population. As has been described in previous studies, more extensive dissection may lead to tissue devascularization and increases in the dead space, which facilitates bacterial growth and ultimately leads to surgical site wound events [21,22].

Early reports investigating the laparoscopic approach to inguinal hernia repair were not favorable [16,23]. In fact, the randomized controlled trial by Neumayer et al. from the Veterans Affairs medical centers [23] concluded that the open technique was superior to the laparoscopic technique for the repair of primary inguinal hernias. The support for the open approach to inguinal hernia repair was initially due to the inexperience with the laparoscopic method for inguinal hernia repair. Nevertheless, as experience accumulated, subsequent studies have shown that the laparoscopic approach is at least equivalent to the open approach for inexperienced surgeons’ hands [24]. Ideally, inguinal hernia repair in the obese patient population should be performed in a way that minimizes the already higher risk of postoperative morbidity while at the same time providing a durable, long-term repair that prevents hernia recurrence [24]. In our study, no hernia recurrence was noted in any patient in both obese and non-obese groups.

The low complication rates in the obese patient subgroup may partly be explained by the careful patient selection for each method (TEP repairs were more preferentially utilized in the obese subgroup) as well as the experience of the surgeons performing the procedures [26]. It should be noted that the present study’s limitations are its retrospective nature and the relatively small number of included patients, which significantly impact the generalizability of the obtained results and potentially suggest that the risk for type I statistical error is present.

## Conclusions

The laparoscopic approach to inguinal hernia repair in obese patients has similar outcomes as an open approach regarding the 30-day events in the hands of experienced surgeons with the advantages of the laparoscopic approach vs. the open one. The choice of repair for an inguinal hernia in obese patients depends on various factors, including the patient's overall health, the size and type of hernia, the surgeon's expertise, and the patient's preferences. We recommend that a surgeon chooses the inguinal hernia repair (open or laparoscopic) that they are most comfortable with in an obese patient.
